# Navigating the future of Alzheimer’s care in Ireland - a service model for disease-modifying therapies in small and medium-sized healthcare systems

**DOI:** 10.1186/s12913-024-11019-7

**Published:** 2024-06-05

**Authors:** Iracema Leroi, Helena Dolphin, Rachel Dinh, Tony Foley, Sean Kennelly, Irina Kinchin, Rónán O’Caoimh, Sean O’Dowd, Laura O’Philbin, Susan O’Reilly, Dominic Trepel, Suzanne Timmons

**Affiliations:** 1 Global Brain Health Institute, School of Medicine, Trinity College Dublin, Lloyd Building, Dublin 2, Dublin, Ireland; 2 Global Brain Health Institute, Dublin, Ireland; 3 HRB-CTN Dementia Trials Ireland, Dublin, Ireland; 4 Institute of Memory and Cognition, Tallaght University Hospital, Dublin, Ireland; 5 Centre for Global Health, Trinity College Dublin, Dublin, Ireland; 6 Department of General Practice, School of Medicine, University College Cork, Cork, Ireland; 7 Department of Geriatric Medicine, Mercy University Hospital, Cork, Ireland; 8 Health Service Executive’s National Dementia Office, Dublin, Ireland; 9 Alzheimer Society Ireland, Dublin, Ireland; 10 Health Services Executive, Dublin, Ireland; 11 Centre for Gerontology and Rehabilitation, University College Cork, Cork, Ireland

**Keywords:** Disease modifying therapies, Alzheimer’s disease, Service readiness, Memory assessment service, Anti-amyloid drugs, Brain health clinic

## Abstract

**Background:**

A new class of antibody-based drug therapy with the potential for disease modification is now available for Alzheimer’s disease (AD). However, the complexity of drug eligibility, administration, cost, and safety of such disease modifying therapies (DMTs) necessitates adopting new treatment and care pathways. A working group was convened in Ireland to consider the implications of, and health system readiness for, DMTs for AD, and to describe a service model for the detection, diagnosis, and management of early AD in the Irish context, providing a template for similar small-medium sized healthcare systems.

**Methods:**

A series of facilitated workshops with a multidisciplinary working group, including Patient and Public Involvement (PPI) members, were undertaken. This informed a series of recommendations for the implementation of new DMTs using an evidence-based conceptual framework for health system readiness based on [1] material resources and structures and [2] human and institutional relationships, values, and norms.

**Results:**

We describe a hub-and-spoke model, which utilises the existing dementia care ecosystem as outlined in Ireland’s Model of Care for Dementia, with Regional Specialist Memory Services (RSMS) acting as central hubs and Memory Assessment and Support Services (MASS) functioning as spokes for less central areas. We provide criteria for DMT referral, eligibility, administration, and ongoing monitoring.

**Conclusions:**

Healthcare systems worldwide are acknowledging the need for advanced clinical pathways for AD, driven by better diagnostics and the emergence of DMTs. Despite facing significant challenges in integrating DMTs into existing care models, the potential for overcoming challenges exists through increased funding, resources, and the development of a structured national treatment network, as proposed in Ireland’s Model of Care for Dementia. This approach offers a replicable blueprint for other healthcare systems with similar scale and complexity.

**Supplementary Information:**

The online version contains supplementary material available at 10.1186/s12913-024-11019-7.

## Background

Alzheimer’s disease (AD), the most prevalent type of dementia, constitutes approximately 70% of all dementia cases globally among people aged 60 and above. Projections suggest that the current 35–40 million individuals affected by AD worldwide will rise to at least 100 million by 2050, with substantial implications for individuals, their families, and healthcare expenditure [1]. Age stands out as the primary risk factor for AD, indicating a heightened vulnerability due to the aging population, and representing a significant gap in medical care [2].

Until 2021, there was no licenced treatment to delay or slow the progression of the neurodegeneration that characterises AD [3]. However, over the past decade, evidence supporting the potential for dementia prevention through risk reduction has increased [4], alongside a growing pipeline of medications with the potential for disease modification [5], targeting the very earliest stages of AD, including mild cognitive impairment (MCI) and the early-stage dementia. Of the current ongoing trials of 126 agents for AD worldwide, about 80% are classified as ‘disease modifying therapties’ (DMTs), as opposed to symptomatic therapies for cognitive enhancement or management of neuropsychiatric symptoms [5]. Current potential DMTs for AD include human monoclonal antibody-based agents targeting beta amyloid.

Recently, regulatory approval was obtained in both the United States of America (USA) and Japan for two anti-amyloid monoclonal antibodies showing potential DMT properties: aducanumab and lecanemab. Aducanumab underwent review by the European Medicines Agency, but did not secure approval for use in Europe. Conversely, lecanemab is currently undergoing regulatory review in Europe. Additionally, recent data have revealed promising results for another DMT candidate, donanemab, which is progressing towards approval. Notably, all anti-amyloid monoclonal antibody DMTs currently approved or on the approval pathway require biomarker confirmation of the diagnosis of AD through demonstrating ‘β-amyloid positivity’, using either PET-ligand neuroimaging, or amyloid-tau ratio cut offs from cerebrospinal fluid, obtained via lumbar puncture [3]. Plasma-derived levels of tau/β-amyloid are not yet approved for clinical use. The early evidence of these monoclonal antibody treatments is associated with potentially high-risk side effects such as brain oedema or microhaemorrhages (i.e., amyloid-associated imaging abnormalities; ARIA) which require serial monitoring with brain MRIs and close clinical follow-up during treatment [6]. This represents a departure in the current approach to AD, warranting the urgent need to ascertain care pathways for these new drugs. Although these medications have yet to receive licencing approval outside of the USA and Japan, a policy analysis on how such DMTs could be incorporated into existing services is also needed.

### Ireland as a case study

In Ireland, a MCI prevalence estimate of 6% of adults over age 60 years is accepted [3], suggesting that about 57,000 of people in this age group may have MCI, although the MCI may not always be due to an underlying neurodegenerative disorder. Nonetheless, this number, together with those with early-stage AD dementia, represents a significant number of people who may benefit from approaches to prevent or delay the onset of dementia. Considering the specific inclusion criteria for the current DMTs, it is estimated that up to 20,000 people might qualify. However, when patients present to memory clinics with subjective memory complaints (SMC), or MCI, they are often discharged back to primary care without further support or intervention. Thus, it is imperative to consider systematic approaches to managing early-stage cognitive decline, since evidence suggests early interventions are associated with larger clinical benefits [7], and foster potential for access to DMT. Details of how the estimates for potentially eligible patients in Ireland were arrived at are outlined in Supplementary Material.

Memory clinics, initially established as tertiary referral medication-management clinics, were introduced to the National Health Service (NHS) in the United Kingdom in the 1990’s, when cognitive-enhancing medications for AD were first licensed. However, memory clinics are now specialist centres that diagnose and treat memory disorders, including dementia. Until recently, Ireland had only 25 such memory clinics, across 13 counties, but many operated less than weekly and were cohorted medical clinics, without a full range of disciplines. Currently, however, driven by Ireland Health Service Executive’s National Dementia Office (NDO), there are plans to expand diagnostic capability significantly over the next five years. This will add new services, as outlined in the recently launched national ‘Model of Care for Dementia in Ireland’ [8].

This new Irish model of diagnosis and care for AD, and other forms of dementia, was informed by the European research project “ACT on Dementia” [9]. The model describes a three-tiered national service, increasing the number of existing local Memory Assessment and Support Services (MASS;level 2), which are supported by primary-care led diagnostic services for low complexity cases (level 1), and by new Regional Specialist Memory Clinics (RSMC; level 3) for higher complexity cases. The RSMC focusses on complex diagnoses, while the MASS provides a full range of services, including brain health and post-diagnostic management and support. Under this new model, neurology, psychiatry of later life and older person’s medicine (medical gerontology) provide integrated services for diagnosis and post-diagnostic care.

The Model of Care for Dementia [8] includes one MASS per local population of 150,000 people (i.e., three Community Health Networks), and a minimum of five RSMCs nationally, with at least two of these based outside Dublin. The first phase of this expansion has taken place, with ten new MASS clinics and two new RSMCs, across the country, funded in 2021 and 2022. Prior to this, existing memory clinics had limited capacity to diagnose, administer and monitor complex new therapies such as DMTs, particularly regarding the anticipated increased need for biomarker ascertainment and safety monitoring including radiological surveillance. Thus, careful consideration regarding the requirement to deliver a new DMT service are outlined below, addressing minimal service requirements.

### Local service audit of the prevalence of patient anti-amyloid treatment eligibility 

Since the prevalence estimates of potential DMT recipients are based on numerous assumptions, we present audit data, including CSF biomarker data collected from 184 patients between 2017 and 2023, from a local MASS in Tallaght University Hospital, Ireland. Of these patients, 39.6% (73/184) were positive for AD biomarkers (low AB-42 and high P-Tau), 25% (46/184) were negative (both AB-42 and P-Tau in normal range), and 35.3% (65/184) were indeterminate (i.e., one of low AB-42 or high P-Tau) [10]. Retrospective case note review was available for 70 CSF-positive patients with AD. Of these, 40 (57%) met potential eligibility criteria for aducanumab therapy by ‘Appropriate Use Criteria’ guidelines [11]. Thus, we can conclude that over half the patients with positive AD biomarkers presenting with prodromal to early-stage AD to a MASS, may be suitable for DMTs based on current treatment indications [12]. We note that patients receiving anti-coagulation therapy generally do not undergo lumbar punctures and thus would not be offered anti-amyloid DMTs.

## Objective

In 2022, the NDO in Ireland convened an Expert Reference Group (ERG) on preparedness for the imminent licensing of new DMTs for AD. Here, we report on the findings of the group’s workshop-style discussions in the form of a blueprint for the implementation of DMTs for prodromal (i.e., MCI) and early-stage dementia due to AD in Ireland. This blueprint extends existing and more detailed guidance on the infrastructure required for the administration of DMTs for AD [11, 13]^1^

## Methods

### Approach and framework

Facilitated workshops were convened by Ireland’s NDO, following a qualitative research model. The workshops were attended by an 11-member multidisciplinary ERG. The aim of the workshops was to scope the current capability and capacity of MASS/RSMC in Ireland, to project demand for DMTs based on Ireland’s population and the current prevalence of AD, and to define system readiness for the introduction of the DMTs. Additionally, potential challenges to developing such a service were identified. The discussions were informed by a new conceptual framework of health system readiness described by Palagyi et al., 2019 [14]. Originally devised to assess system preparedness for emerging infectious diseases, the framework, consisting of six core constructs, also has utility for the proposed DMT service. Four of the constructs focus on material resources and structures (i.e., system ‘hardware’), including (i) Surveillance, (ii) Infrastructure and medical supplies, (iii) Workforce, and (iv) Communication mechanisms; and two constructs focus on human and institutional relationships, values and norms (i.e. system ‘software’), including (i) Governance, and (ii) Trust.

The ERG consisted of geographically dispersed individuals experienced in various facets of AD care in Ireland, including an academic geriatric psychiatrist, a specialist trainee geriatrician, two consultant geriatricians who are memory clinic leads, a consultant cognitive neurologist, a GP with special interest in cognitive health, two health economists, a memory clinic specialist nurse practitioner, and a representative from the HSE and third sector partner, the Alzheimer Society of Ireland, representing people with lived experience of AD and their care partners. Five workshops were held serially, either face-to-face or remotely and were facilitated by a chairperson.

### Data collection and analysis

Workshops were recorded and field notes obtained for subsequent narrative and descriptive analysis. Modelling of projected demand for DMTs was informed by current national and international prevalence data of prodromal (MCI) and early-stage dementia due to AD, AD biomarker positivity, and other factors relevant for DMT eligibility.

## Results

### Purpose of an early diagnosis and DMT intervention service for AD

The ERG agreed that a new DMT service for AD should be fully embedded in existing or proposed RSMC units. As such, it would be an additional layer of service integrated into the pathway for diagnosis, initial care planning, and post-diagnostic interventions. Patients would retain close ties with their local MASS to access brain health support, and the full range of post-diagnostic interventions, in parallel with the provision of DMTs. Table 1 outlines the specific purposes of the DMT service.

**Table 1:** Components of a disease modifying therapy (DMT) service for Alzheimer’s disease in Ireland or similarly structured healthcare system

Aim of a DMT service for Alzheimer disease	
• To diagnose or confirm the diagnosis of patients with early-stage cognitive decline due to AD or other neurodegenerative conditions, according to updated clinico-biologic diagnostic classifications [27].	
• To identify patients eligible for DMTs and offer relevant information regarding the risk and benefit of the DMT, along with appropriate support to the patient in making this decision.	
• For those opting for and eligible for DMTs, to administer the treatment according to best practice, based on consensus- and evidence-based Appropriate Use Recommendations [6, 11].	
• To monitor safety as long the patient is receiving DMTs (i.e., serial MRIs, adverse event screening).	
• To monitor outcomes and/or response to treatment, applying clinical decision-making regarding effectiveness or usefulness of the treatment.	
• To ascertain and counsel patients and families regarding termination of treatment and appropriate next steps.	
• To link with local MASS to ensure integrated care including access to brain health support, cognitive therapies, interventions for non-cognitive symptoms, information, support, etc., as appropriate.	
**Referral criteria for a DMT service for Alzheimer’s disease**	
• Adult age (above 18 and ≤ 85)	
• Self- or informant-reported cognitive complaint	
• Progressive objective cognitive impairment meeting criteria for MCI, or mild dementia due to AD (i.e., preserved or relatively preserved independence in functional abilities, defined by ‘activities of daily living’)	
**Minimal service capacity requirements for a DMT service**	
Capacity to determine biomarker status	For amyloid-based DMTs, there is a requirement for biomarker-based confirmation of amyloid status using either amyloid-β PET imaging (for in vivo detection of fibrillar plaques, a core neuropathological feature of AD), or reduced soluble amyloid-β in CSF. In the near future, the use of less resource-intensive plasma biomarkers (blood-based markers) could facilitate this [28].
Medication administration infrastructure	It is likely that the first wave of AD DMT to be licenced for use in Ireland will require regular intravenous infusions. Memory services in Ireland do not currently provide infusions as an intrinsic part of the service. However, many existing memory clinics, cognitive neurology clinics and the in-development/proposed MASS and RSMC are co-located with neurology services that administer infusion-based ‘biologics’ for multiple sclerosis and other neuroinflammatory disorders, and/or older person services that routinely provide other infusions (such as blood, iron, bisphosphonate infusions, etc.).
MRI neuroimaging	This is required for diagnostic verification and to confirm eligibility for DMTs (by ruling out existing (micro) haemorrhages or other structural pathology that would predispose to haemorrhage) and for ongoing safety monitoring post-treatment administration. The emergence of amyloid-related imaging abnormalities (ARIA) [29] seen as MRI signal abnormalities involving oedematous (ARIA-E) and haemorrhagic changes in the brain (ARIA-H), related to target engagement of amyloid treatments, requires regular monitoring through serial MRIs throughout the period of treatment. In addition, there is a need for prompt access to neuroimaging, including MRI, should the person develop concerning neurological symptoms, with the urgency and choice of neuroimaging related to the clinical presentation, but ideally requiring weekend availability of MRI.
Links to brain health services	The new emphasis on supporting healthy brain lifestyles, with a view to prevention, is a key element to a DMT service for [1] referrals for people in the earliest stages of cognitive decline; and [2] ongoing multidomain intervention to delay progression, even when taking a DMT. Various clinic- or community-based models of brain health services now exist.
**Components of a DMT service for Alzheimer’s disease**	
***1. Assessment domains to ascertain risk-based ‘streams’ on intervention***:	
**Clinical and lifestyle profile**:	Potentially key reversible risk factors need to be ascertained to inform risk-based decision making, and may include evaluations of vascular health status (e.g. diabetes, hypertension), sensory function (i.e. hearing and vision), mental health status (e.g. depression), social circumstances (e.g. isolation, loneliness, support network [30] and lifestyle profile (e.g. physical and cognitve activity, diet).
**Cognitive profile and staging**:	More detailed neuropsychological testing as per the Model of Care (https://www.ncbi.nlm.nih.gov/pmc/articles/PMC4635684/). These will support identification of the ‘at risk’ cohort (i.e. detecting a ‘hippocampal signature’ characteristic of amnestic MCI, indicative of possible or probable AD and progression to dementia). The CDR scale [31] will be applied to ascertain stage of cognitive decline.
**Behavioural and functional profiling**:	In the prodromal stage, subtle behavioural and functional changes may be present and be harbingers of later decline, assessed using tests such as the Amsterdam IADL Questionnaire [32] for functional profiling and the Mild Behavioral Impairment Checklist [33], both of which are sensitive for early state dementia.
**Biomarker detection**:	Alzheimer-related biomarkers include β amyloid (A) deposition, pathologic tau (T), and neurodegeneration (N), or the ATN classification ‘biological’ diagnosis of AD is not yet widely used clinically. However, the UK’s NICE guidance (2018) recommends lumbar puncture for the diagnosis of AD in people with MCI whose diagnosis may be unclear [34].
	Genetic testing for AD risk using the apolipoprotein (APOE) genotype is not widely recommended in clinical settings despite being more readily available. Practice guidelines for the use of APOE testing have been published [35].
	• β amyloid (A) and pathological tau (T) deposition
	These can be detected either by CSF lumbar puncture or PET scans with amyloid or tau tracers. Levels of p-tau are elevated in the CSF of patients with AD and other neurological conditions as compared with healthy controls. A recent meta-analysis by Olsson, et al. [36] recommends using CSF Aß42, T-tau, p-tau and NFL levels as a panel of diagnostic biomarkers for AD in both clinical practice and research, if available.
	• Neurodegeneration (N)
	This can be detected by structural neuroimaging using magnetic resonance imaging (MRI) to detect such changes as medial temporal atrophy, suggestive of AD and changes indicative of cerebrovascular disease (quantified using Fazekas scores), as well as FGD-PET neuroimaging. Additionally, detection of elevated CSF levels of neurofilament light chain (NFL) protein, a marker for neuroaxonal damage, may add to the sensitivity and specificity of the presence of neurodegeneration.
***2. Eligibility criteria for receiving a DMT***:	
**Inclusion criteria**	•Diagnosis of Alzheimer disease, as per accepted criteria
	•MCI or mild dementia stage, as determined by CDR (global) 0.5-1.0 at time of consideration for DMT treatment
	•For anti-amyloid DMTs: Amyloid positivity on CSF biomarker analysis or amyloid-PET scan
	•Functionally independent in essential ADLs
	•Living independently or with a care partner
	•Age 50–85
	•Consenting to ApoE genotype testing
	•MRI brain within past one year with evidence supporting the diagnosis of AD
	•Co-prescription of current therapies (cholinesterase inhibitors) will be acceptable
	•Stable medical, cardiovascular and psychiatric conditions as determined by prescribing clinician
**Exclusion criteria**:	•Any evidence on MRI of acute haemorrhage, or > four microhaemorrhages, or infarct > 1.5 cm, or areas of superficial siderosis, or diffuse white matter disease
	•For anti-amyloid DMTs: If taking regular oral anticoagulant (due to ARIA risk)
	•Comorbid illness will need to be accounted for as determined by the prescribing clinician.
	•Relevant non-AD neurological disorders as determined by prescribing physician should be excluded
***3. Intervention administration***:	
**Medication rationalization**	Benzodiazepines, opioid analgesics, certain psychotropics, and medications with high anticholinergic loads should be reviewed and rationalized to ensure they are not contributory to cognitive impairment or decline.
**Managing co-morbidity**	Addressing medical comorbidity, particularly vascular risks, sensory functioning, and other physical causes of cognitive decline need to be addressed, establishing a baseline for ongoing monitoring of medical risk factors and potential modification.
	This may be provided as part of the service providing the DMT, or may be addressed locally by a MASS if the MASS is not providing the DMT.
**Pharmacological interventions**	As the therapeutic options expand, interventions will likely involve different mechanisms of actions such as anti-amyloid and anti-tau antibody-based agents, and anti-inflammatory agents inhibiting pathological cascades.
**Non-pharmacological interventions**	Multi-modal intervention care plans such as risk modification [4] and non-pharmacologic cognition-based interventions have demonstrated improvements in memory in healthy older people and people with MCI [37]
	These non-pharmacological interventions may be offered by the DMT clinic or, alternatively and more likely, by the local MASS in the brain health pathway.
***4. Ongoing care and monitoring during DMT administration period***:	
**Periodic MRI Monitoring**	ARIA identification will be either: in an asymptomatic person via regular scheduled MRI; or due to symptoms, which will be monitored by the prescribing clinician. Management of ARIA will be protocol-dependent, depending on extent of ARIA and symptoms.
**Cessation of Therapy**	Cessation of therapy should occur if: [1] the patient has any allergic reaction; [2] in the case of a new or advancing medical illness that may place the risk of AD DMT treatment as outweighing the benefit; [3] adverse events, including ARIA-H or ARIA-E; [4] non-adherence with safety monitoring protocols (i.e. serial MRIs); [5] patient preference; and [6] non-adherence with medication administration protocols. If the person has a Clinical Dementia Rating scale global scale of > 2.0 (this should be made on two assessments that are temporally remote and should employ the clinical judgement of the prescribing clinician).

### Material resources and structures (‘system hardware’)

#### Surveillance to detect early disease

Early detection and monitoring of progression of cognitive decline in the earliest stages (prodromal AD or early-stage dementia due to AD) should be a key element of DMT preparedness, supported by evidence of the potential effectiveness of disease modifying approaches prior to moderate- or advanced-stage dementia. This requires early detection at the clinical level, but also greater public awareness and health literacy for timely help seeking.

##### Early clinical detection

Ideally, blood-based biomarkers, available in primary care, would be available to enable early detection. Since such biomarkers are as yet not widely available in clinical settings, raising awareness amongst the public and primary care providers needs to take place to foster early clinical detection. Importantly, initiatives to introduce widespread cognitive screening for older people in the UK were not supported [15] due to the risk of over-diagnosis and the lack of meaningful interventions in the early stages of AD. With the advent of DMTs, such screening initiatives may need to be revisited.

##### Public awareness and health literacy needs

To enable successful implementation of the new DMTs, it is important to harness political willpower by presenting DMTs as a public health investment, rather than a cost, with a clear narrative around economic savings that could accrue from delaying conversion to or progression of dementia. Economic data specific to Ireland is currently not available. However, a model developed in the USA to assess the impact of DMTs on AD highlights substantial benefits [16]. It predicts that a 5-year delay in the onset of AD, leading to a 25% reduction in its prevalence by 2050, could result in cumulative savings of over $3 trillion between 2022 and 2050 [16].

Research at a national scale that captures public perception, expectations, and concerns about DMTs is also required, and any public health literacy campaign should be designed in collaboration with stakeholders, including PPI contributors, capitalising on existing resources (e.g., Ireland’s ‘Understand Together’ dementia campaign). Finally, public health messaging should highlight the importance of an early diagnosis, while managing expectations regarding the efficacy and narrow eligibility criteria for DMTs.

#### Infrastructure and provision for an early diagnosis and intervention service for AD

**Fig. 1:**
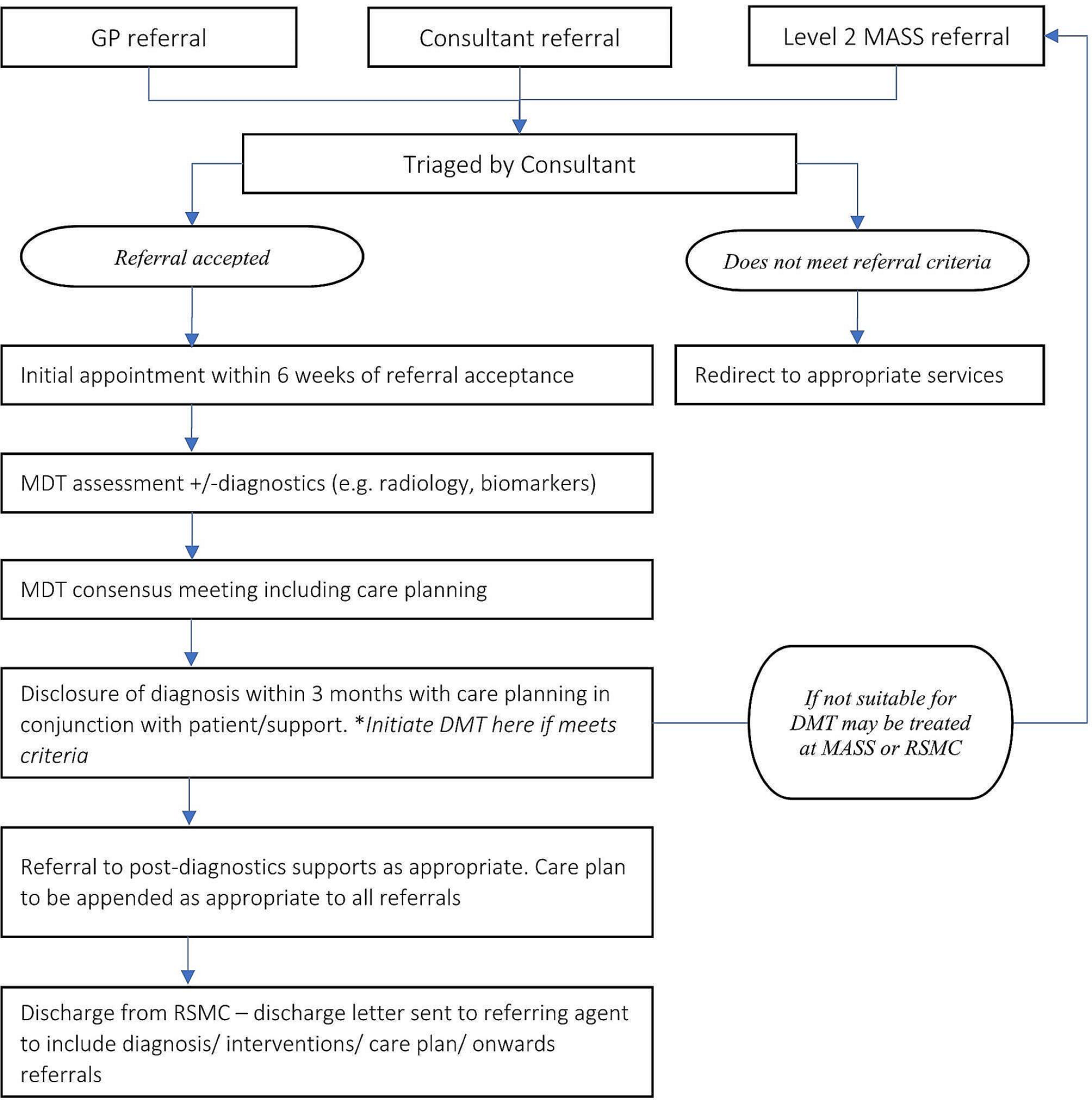
Schematic of infrastructure and provision for an early diagnosis and intervention service for Alzheimer’s’ Disease (AD) in Ireland

The EGR debated the utility of a distributed versus centralised service model for DMT delivery. One commonly used model in Irish healthcare is a ‘hub-and-spoke’ model (e.g., in hyper-acute stroke care), which can provide a practical compromise, as shown in Fig. 1. This entails one or more RSMC acting as a central hub, ideally in different regions of the country, and local MASS acting as spokes across the country. Rolling out DMT provision in one or two highly resourced pilot sites in the first instance, rather than starting with a fully distributed service, would ascertain eligibility rates and DMTs uptake, and develop experience around the administration and monitoring of DMTs. In the meantime, existing MASS services (spokes) will see relatively small numbers of people potentially eligible for DMTs. Thus, local referral pathways will be needed from these services to a nearby RSMC, or a MASS that has elected to provide DMTs in the first wave, so that the person can have detailed eligibility assessment and access to a DMT.

##### Minimal service capacity to deliver DMT

The minimal service capacity requirements to deliver DMTs are detailed in Table 1 and include: [1] capacity to ascertain biomarker status for AD; [2] medication administration infrastructure (e.g., infusion facilities); [3] sufficient MRI neuroimaging capacity for both diagnosis and monitoring; and [4] links to brain health pathways and post-diagnostic support.

##### Patient referral criteria to the DMT service

It was agreed that the service providing access to anti-amyloid DMTs would identify its own pool of eligible patients within its MASS/RSMC remit, as well as accepting referrals from other MASS, diverting a patient directly to the MASS/RSMC or performing an initial assessment/full diagnosis and disclosure prior to referral. Suggested patient referral criteria to the DMT service are listed in Table 1. Patients would not be accepted into the service if they had non-degenerative cognitive impairment due to another identified cause at the point of referral (e.g., depression, alcohol, or drug misuse, Vitamin B12 deficiency, thyroid disease, and others). If cognitive complaints persisted after effective treatment of a primary illness, then referral could be considered.

##### Components of the DMT intervention pathway

The DMT arm of a memory service would have three main components: [1] *assessment and diagnosis;* [2] *intervention administration;* and [3] *ongoing monitoring and care*. These components would run parallel to a brain health clinic model offered by local MASS services and are also outlined in Table 1, along with a list of eligibility criteria for DMT, which align broadly with the inclusion criteria of the relevant clinical trials of these same DMT [3]. It is noted that at the point of referral to local MASS, it is expected that a basic medical and cognitive work-up will have been completed in primary care to rule out reversible causes for cognitive complaints (e.g., alcohol, or drug misuse, vitamin B12 deficiency, thyroid disease, and others) and to establish that the patient is in the prodromal or mild dementia stage. Once a referral has been accepted, a detailed assessment including biomarkers would be undertaken, as summarized in Table 1 under the first component of the service, ‘*Assessment domains*. This will ascertain diagnostic sub-type and prognosis of early-stage cognitive decline. The assessment would include clinical, lifestyle, behavioural, functional, and cognitive assessments as well as biomarker detection. Key biomarkers, as recommended under the International Working Group (IWG-2) criteria for AD [6] are briefly outlined in Table 1. Specifically, these criteria define the clinical phenotypes of AD (typical or atypical), integrating pathophysiological biomarker consistent with the presence of AD into the diagnostic process [17]. The use of biomarkers in the diagnosis of AD in the prodromal stage has altered the characterization of AD from being a syndrome-based diagnosis to a biologically-based diagnosis. A biomarker-based approach will support more personalized therapeutic approaches to the prevention of aging-related brain disorders, taking individual biological, genetic and cognitive profiles into account [18].

The outcome of the assessment will enable patients to be assigned to one of three risk-based ‘streams’: [1] begin the DMT care pathway; [2] be refered to local MASS for brain health pathway or relevant care for non-AD neurodegenerative disorders; and/or [3] be referred for a research study. Details of an approach to brain health management along with the new DMTs has been outlined elsewhere [13]. Under the ‘*intervention administration*’ domain, guidelines for rationalization of prescribed medications, managing comorbidity, and pharmacological and non-pharmacological interventions are outlined, and under the ‘*ongoing monitoring and care*’ domain, guidelines for periodic MRI monitoring and possible cessation of therapy are listed. Coordination of care is an important, yet challenging, issue. Ideally, an ‘early diagnosis navigator’ is needed to ensure all patients receive optimal care and foster a pathway back to the main clinic should they be diverted down an alternative path, such as research, brain health, or DMT.

#### Workforce: roles and education

##### Roles and expertise required

The availability of frontline healthcare workers in sufficient numbers and with appropriate training and expertise to administer and monitor DMTs is a key feature. Whilst the specialist assessments and initial interventions will be undertaken by members of the core MASS/RSMC team, they will link in with a range of services and providers, both internal and external, as per the Model of Care for Dementia in Ireland. The MASS/RSMC team should meet regularly in person or virtually for multidisciplinary team (MDT) meetings. Finally,, there are additional MDT roles specifically for DMT provision, which exceed the role of the MDT as outlined in the Model of Care for Dementia (see Table 2).

**Table 2:** Required workforce and role description for a DMT service for Alzheimer’s disease in Ireland or a similar healthcare system

Role for DMT Provision	Role Description
Consultant (neurology, psychiatry of later life, or geriatric medicine)	Be the clinical and prescribing lead, with links to research and audit programs
Specialist trainee	Support the consultant role and provide lumbar puncture access
Nurse specialist	Oversee infusions, monitor follow-up safety scheduling, and oversee the assessment response and safety monitoring
Clinical psychologist	Support any additional diagnostic workup through neuropsychological assessments, if needed, and assist in treatment termination decisions
Neuroimaging specialist	Provide diagnostic support through MRI and PET imaging and other neuroimaging modalities as required to determine eligibility and for safety monitoring
Allied health professional (occupational therapy, speech and language therapist, etc.)	Support additional diagnostic workup and disclosure as required for the DMT eligibility assessment, and support practical solutions to facilitate DMT access if required (accessibility, communication difficulties, etc.)
Social Worker	Support solutions to barriers that may exist to accessing the infusion (medical card eligibility, transportation solutions, etc.)
Administrator	Ensure scheduling of patients and close links with local MASS

##### Education and training of workforce

Delivery of an accurate diagnosis, identification of suitable treatment candidates, and monitoring of ongoing DMTs will require a standardised training program. Clinicians from multiple specialities (older person’s medicine, psychiatry of later life, neurology, general practice) involved in the work-up and diagnosis of dementia will require training delivered via the Royal College of Physicians Ireland, Irish College of General Practitioners, and the Irish College of Psychiatry. This would involve the application of AD biomarkers and therapeutic indications for prescribing DMTs. Radiologists will require training in the interpretation of evolving imaging modalities supporting the diagnosis of AD and other dementia subtypes. Training for clinicians on the identification of adverse drug reactions (e.g., ARIA) will be required and delivered as an ongoing iterative process as the field advances.

#### Communication mechanisms

Considering the hub-and-spoke model across the geography of Ireland, robust, timely, and standardized communication between the hub and spokes is necessary for ensuring patient safety. Additionally, the use of visual representations of the health system structure, such as the one detailed in Fig. 1, will facilitate decision-making and preparedness among users and policy makers.

### Human and institutional relationships, values, and norms (i.e., system ‘software’)

#### Governance: need for a national AD DMT patient registry

Governance emphasizes the creation and monitoring of the rules that govern the supply and demand of health services. There should be a national AD DMT patient registry for accurate patient safety monitoring at a national level, and to support service planning. These data can be recorded along with other mandatory data from the developing MASS and RSMC (as an adjunct to the already proposed minimum dataset), but there may be additional monitoring requirements, dictated by Ireland Health Products Regulatory Agency (HPRA). It should be noted, however, that establishing a patient treatment registry is complex and requires significant resources, both human and financial. Challenges to consider in setting up a registry include ensuring the quality of the data, sustainability, governance, financing, and data protection. It is very likely that the pharmaceutical industry will play an important part in the set up and support of a registry, possibly linked to their licensing agreement [19].

#### Trust

Trust is a fundamental component of health system preparedness, incorporating both interpersonal trust (between patient and provider) and institutional trust (between individuals and the health system or government) [20]. Furthermore, trust is a prerequisite for health system resilience, particularly as new paradigms of care are being introduced. Health systems that have the trust of the population and political leaders by providing quality services prior to a health urgency have greater resilience [21].

### Current provision of services in Ireland that are ‘DMT ready’ and the projected need

If and when a DMT such as lecanemab gains approval for use Ireland, the additional demand on services would be significant, including additional diagnostic services for biomarker detection (i.e., lumbar puncture, ligand-based PET scans), drug administration (i.e., infusion facilities such as day hospitals), and treatment-related safety monitoring capacity (i.e. serial brain MRIs). Considering these minimum requirements to offer an infusion-based DMT in Ireland, it is likely that it could only be offered by a limited number of centres in Ireland.

#### Projected neuroimaging requirements

Currently, an MRI scan is the preferred imaging modality in a memory service to assist with early diagnosis and detection of subcortical vascular changes [12]. Since the amyloid-based DMTs are associated with ARIA, ongoing monitoring for those on treatment would be needed. Based on the estimated prevalence figures above, and an anticipated need for at least three routine monitoring scans per person, this would necessitate 10,140 − 65,640 scheduled MRIs.^2^ Additionally, about 40% of patients on DMTs may require up to three additional MRIs due to the actual development of ARIA or neurological sequelae, equating to 4056-26,656 non-routine MRI scans, so that the total early requirement for additional MRI scans would be 14,196 − 92,296 scans, required over the first two to three years post-licencing.^3^ Steady-state MRI demand by 2030 is estimated to be 5,720 − 30,879 scans per annum.

### Challenges to delivery of DMT for Alzheimer’s in Ireland

Table 3 summaries several areas that may pose both structural and ethical challenges to these new treatments for AD in Ireland.

**Table 3:** Structural and ethical challenges to delivering a DMT service for Alzheimer’s disease in Ireland or similar healthcare system

Structural challenges:
• Historic lack of early referral to services, resulting in patients being ‘out of window’ for DMT treatment
• Currently insufficient provision of memory services nationally, noting that the forthcoming model of care, if funded, will alleviate much of this challenge
• Infrequent undertaking of biomarker testing via CSF analysis or PET scanning in current memory services, such that services will need to be scaled up involving staff training
• Insufficient MRI scan capacity for image acquisition and insufficient dedicated neuroradiologists in Ireland for the required diagnostic imaging and for the monitoring necessary for safe administration of DMTs
• Infrastructural hospital space, access to infusion suites and trained staff to administer these IV therapies may be a challenge, especially during surges in acute hospital unscheduled care demand (less of an issue if DMT administration is provided in MASS that are based in community ambulatory care hubs)
• The need for appropriate resource allocation for setting up and monitoring the DMT aspects of the dementia patient registry
**Ethical challenges**:
• Age cut offs which raise the consideration of a patient outside of the window i.e., a 49-year-old with AD signature on CSF and whether they would be accepted for treatment
• Exclusion of those with subjective cognitive complaints (only those with object loss in these domains i.e., a global score of 0.5-1.0 were included in the seminal trials)
• Planning for those who have cognitive symptoms but *do not have AD* pathology detected via CSF or PET scanning, i.e., those with FTLD, tauopathies, PSP, DLB etc.
• *Cessation of therapy*, specifically when during the disease course to stop, how to ensure a drop in cognition is not due to an underlying illness, or how to weigh up the utility of continuing this therapy if new diseases are diagnosed
• *Diversion of funding* from existing care models and community-based supports towards a group of persons for whom DMTs may be suitable
• *AD phenocopies with negative biomarkers.* The **“**AD phenocopy” is prevalent in older and APOE ε4 negative patients and may be due to a combination of age-related pathologies of the limbic system, thus presenting with an AD-like amnestic profile and will not be suitable for AD-directed DMTs. Additionally, a proportion of patients may have false-negative PET scans, reflecting lack of sensitivity in detecting advanced amyloid pathology, Alternative biomarker detection (i.e. CSF) can obviate against this.
• *ApoE genotype* testing should be part of the work up for those under consideration for AD DMTs given the higher likelihood of adverse events for ApoE4 allele carriers, and the ethical consideration for undertaking (including the optimal timing) and disclosing the results of genotype testing for both the person and their family will need to be considered.

## Discussion

Using a conceptual model of health system preparedness, we have presented a proposal for the delivery of anti-amyloid DMTs for AD, integrated within a hub-and-spoke model as part of the newly launched Model of Care for Dementia in Ireland. We acknowledge that the implementation of any healthcare model requires both system ‘hardware’ (tangible components such as infrastructure and workforce) and system ‘software’ (intangible components such as human values and power dynamics) [22]. As such, our current systems would likely face challenges in implementing DMT services without active efforts towards building individual and institutional trust and obtaining additional resources. Therefore, we anticipate that the full evolution to a national network of MASS with supporting RSMC, will take approximately three to five years to achieve and is subject to funding.

Moreover, while DMTs for AD offer the potential to revolutionize the management of the disease, it’s crucial to approach their development and implementation with careful consideration of both their benefits and limitations. Potential benefits include slowing of disease progression, improved quality of life, and delayed institutionalisation, along with associated economic benefits. However, these potential benefits need to be weighed against limitations such as moderate effectiveness of the drugs, potentially serious side effects, and ethical considerations such as treatment accessibility and affordability, and ascertaining what is meaningful to patients and their families [23].

### Actions for the future

It will be important for healthcare systems to remain abreast of developments in order to offer those affected by AD the latest and most effective therapies. Part of this effort involves playing a role in drug discovery and evaluation. Recently, the Irish Health Research Board’s Clinical Trials Network funded a 5-year dementia clinical trials’ infrastructure development program, Dementia Trials Ireland, to grow Ireland’s capacity to conduct dementia trials [24]. In addition, over the past five years, there has been an additional focus on risk factor modification through lifestyle changes for prevention of dementia [4]. This approach is critical, and Ireland needs to keep pace with the rest of the world in addressing this issue. Finally, it is important to highlight inherent structural risks to the implementation of the proposed model. The future delivery of DMTs is complicated by long-standing capacity constraints within the Irish healthcare system, insufficient universal primary care coverage, and growing waiting lists [25]. As structural and policy reform continues as manifested by the publishing of National Dementia Strategy (2014) the establishment of the NDO (2017), and the launch of the Model of Care for Dementia (2023), this must be underpinned by targeted spending.

## Limitations

The analysis was based on a consensus exercise including mostly healthcare professionals supported by consultation with healthcare recipients. Ideally, people with lived experience of AD and their care partners should be included in the main consensus process. Additionally, our data on the numbers of potential DMT recipients are broad estimates. To date, we lack robust epidemiological data on biomarker-positive individuals who may be eligible for the drugs. Additionally, the system readiness model we used is limited by the lack of consideration of funding, which is critical to future developments and implementation of DMTs. Moreover, risk-prediction modelling is not considered in the model, and although it is an active area of research, it has not been clinically implemented on a wide scale.

Other limitations include the dynamic nature of healthcare policies and the need to continuously adapt the system to consider changes in healthcare polices, funding structures and regulatory frameworks. Changes in population demographics also need to be considered, along with technological and IT infrastructure demands. Finally, other models besides the hub-and-spoke model may have merit and could provide alternative approaches to addressing this impending change in dementia care.

## Summary

Healthcare systems around the world are recognising the urgent need for next-generation clinical care pathways for AD, prompted by enhanced diagnostics and the emergence of DMTs. Concerns about the ability of existing delivery models to introduce such therapies efficiently and equitably have been highlighted in several European countries [26]. Echoing European colleagues, Ireland’s healthcare system faces challenges to fully incorporate the prescription of DMTs into routine clinical pathways. However, these challenges may be overcome with additional resources, financial investment, and evolution to a structured, national treatment network, as envisaged by the recently launched ‘Model of Care for Dementia in Ireland’ [8]. We suggest that the blueprint outlined in this paper, developed in conjunction with facilitated workshops, including PPI representatives, is replicable for other healthcare systems of comparable size and scope in Europe and further afield.

### Electronic supplementary material

Below is the link to the electronic supplementary material.

Supplementary Material 1

## Data Availability

All data generated or analysed during this study are included in this published article.

## Footnotes

1 At the time of writing, whilst Biogen was granted an accelerated approval for their monoclonal antibody (aducanumab) by the US food and Drug Administration (FDA), the European Medicines Agency (EMA) rejected the marketing authorisation application based on results from the two-phase III clinical trials.

2 As detailed earlier, we anticipate 3,380 to 21,880 people with prevalent AD-MCI or mild AD may proceed to DMT after licencing. As at least 3 monitoring scans are required per person, this equates to 10,140 − 65,640 additional “routine” MRIs. In addition, 40% of these are expected to develop ARIAs (from trial data), where up to 3 additional MRIs would be required (depending on speed of resolution), equating to 4,056 − 26,656 non-routine MRI scans required over a specified period. Once the existing pool of “prevalent” people is treated, steady-state MRI demand will be due to incident AD-MCI and mild AD. Based on the projections for an increased population with these two conditions, and also increased uptake of DMTs within this eligible pool, we estimate 1,362 to 7,350 people proceeding to DMT (see workings in footnote 2). This equates to 4,086 − 22,050 routine MRIs (3 per person), and 1634-8,820 non-routine MRIs for ARIAs (40% occurrence of ARIAs, and 3 scans per person for ARIA monitoring). Thus, the total annual MRI requirement by 2030 is 5720-30,879.
