# Supplementary material for: Navigating the future of Alzheimer’s care in Ireland - a service model for disease-modifying therapies in small and medium-sized healthcare systems
Source: BMC Health Serv Res. 2024 Jun 5;24:705. doi: 10.1186/s12913-024-11019-7 (PMC11151472; doi:10.1186/s12913-024-11019-7)
Supplement: Supplementary file 1 — Supplementary Material 1 [file 12913_2024_11019_MOESM1_ESM.docx]

Of the estimated 64,000 people living with dementia in Ireland, between 28,400 to 40,900 are aged 50-85 (depending on the exact age-stratified prevalence rate applied) [1]. Within this age range cohort, it is estimated that **2,400-14,300** have mild, AD-type dementia, potentially a target for the anti-amyloid DMTs [1]. This estimate comes from the statistic that 58% of all those with AD dementia are in early-stage dementia (CDR 0.5-1.0 range) = **22,272** cases and 22% of all cases with dementia are amyloid positive = 4,454 with mild amyloid-positive dementia.

People below the age of 50 are more likely to have non-AD causes of dementia, and those over age 85 may not be able or willing to undergo monthly infusions, with higher inherent risks of intracerebral bleeding. Furthermore, within the “potentially eligible” cohort of people with mild AD-type dementia, many will not have presented for diagnosis during the eligible time-window, will choose not to undergo infusions, or will have a contraindication or other comorbidities making DMT-treatment inappropriate. Thus, we estimate that only 10%-20% of the overall potentially eligible pool might actually proceed to infusion therapy, i.e. **240-2,860** people in Ireland [2]. The range presented applies the 10% rate to 2,400 people and the 20% rate to 14300 people. (A supporting estimate is that only 13% of people present to memory services when in the very early stages of memory impairment).

There are an additional estimated 68,000 to 126,790 people aged 50-85 years with MCI in Ireland (depending on the exact age-stratified prevalence applied). These numbers were calculated in the following way: applying the COSMIC MCI age-stratified prevalence rates (4.5% 60-69 years; 5.8% 70-79; 7.1% 80-89; and extrapolating to a 2.9% prevalence for 50-54 and 3.5% for 55-59 years using the slope of the line), there are 68,002 people with MCI aged 50-85 in Ireland. Applying the AAN rate (6.7% for ages 60–64, 8.4% for 65–69, 10.1% for 70–74, 14.8% for 75–79, 25.2% for 80–84; and extrapolating to a 3.5% prevalence for 50-54 and 5% for 55-59 years using the slope of the line), there are 126,787 people with MCI aged 50-85 in Ireland.

Within this cohort, 31,388 to 95,090 are estimated to have prodromal AD (again, depending on the exact age-stratified or blanket prevalence rates applied). Applying Jansen’s age-stratified AD prevalence within MCI rates (25% at 47.5-52.4 years; 27% at 52.5-57.4 years; 39% at 57.5-62.4 years;45.5% at 62.5-67.4 years; 54.5% at 67.6-72.4 years; 57.2% at 72.5-77.4 years; and 62.1% at 77.5-82.4 years) to the COSMIC estimated-MCI prevalence (68,002) and the AAN estimated AD-MCI prevalence (126,787), there are 31,388 to 62,843 people with AD-MCI aged 50-85 in Ireland.

Applying a cruder “75% of MCI is due to AD” rate, the corresponding figures are 51,000 for the COSMIC-derived estimate and 95,090 for the AAN-derived estimate. Thus, the prevalence appears to lie between 31,388 and 95,090 taking the lowest and highest estimates.

If only 10%-20% will proceed to infusion therapy, as per our previous assumptions, this equates to **3,139 to 19,018** people who would require infusion. 3,139 to 19,018 range was found by applying the 10% rate to the lower estimate (31,388) and the 20% to the higher estimate (95,090).

The demand may be at the lower end of the range as most people with MCI in Ireland do not receive a clinical diagnosis. Thus, the total number, between eligible people with AD-dementia (240 to 2860 people) and prodromal AD (MCI due to AD; 3,139 to 19,018 people), is estimated at ***3,380 to 21,880*** people potentially proceeding to DMT infusion therapy within the first few years of DMT being available in Ireland, expecting the figure to be closer to the lower estimate. The 3,380 to 21,880 estimate was found by adding the two lower and the two higher estimates (bold font above) together: 240 + 3139 = 3379 and 2860 + 19,018 = 21,878.

Thereafter, DMT services need to provide a service for people with incident prodromal AD and mild stage dementia due to AD, crudely estimated at 10% of the annual prevalence figure, or 3,380-10,940 people per annum. However, an additional 3% per annum incidence increase due to population ageing (e.g. the annual incidence would rise to somewhere between 4,543 and 14,703 by the early 2030s) needs to be considered. The 2030s incidence is calculated by applying a 3% compound increase over 10 years. In addition, as DMT infusion therapy becomes more available in Ireland in the foreseeable future, the proportion of eligible people choosing to proceed, and the confidence of diagnosing services to recommend treatment will likely rise. Thus, it can be postulated that eligibility for treatment might increase to perhaps 30-50%, which could mean an additional 1,363 to 7,351 people requiring DMT infusions per annum by 2030. The range represents 30% of the lower incidence estimate (4543) and 50% of the higher incidence estimate (14,703).

**References:**

1. Carney, P., E. O’ Shea, and T. Pierse, *Estimates of the prevalence, incidence and severity of dementia in Ireland.* Irish Journal of Psychological Medicine, 2019. **36**(2): p. 129-137.

2. Knopman, D.S., et al., *Mild Cognitive Impairment and Dementia Prevalence: The Atherosclerosis Risk in Communities Neurocognitive Study (ARIC-NCS).* Alzheimers Dement (Amst), 2016. **2**: p. 1-11.
